# Supplementary material for: Risk factors of papillary thyroid microcarcinoma that predispose patients to local recurrence
Source: PLoS One. 2020 Dec 31;15(12):e0244930. doi: 10.1371/journal.pone.0244930 (PMC7775061; doi:10.1371/journal.pone.0244930)
Supplement: Attachment — Submitted filename: Response to Reviewers.doc [file pone.0244930.s002.doc]

***Plos One* Editorial Board**	November 25, 2020

Dear Editor and Reviewers,

First, we truly appreciate the opportunity to resubmit our revised manuscript. Thank you very much for considering our manuscript for potential publication in *Plos One*.

We would also like to thank you for the very thorough reviews, professional advice and constructive criticism, which were highly valuable for improving our paper. All suggestions regarding changes and improvements were very helpful to us, and we revised the manuscript according to the recommendations provided in the reviews. All changed and deleted portions of the manuscript are indicated with *track changes.* According to the reviewers’ instructions, we have corrected our manuscript point-by-point as follows.

Regarding the responses to the concrete comments by the reviewers, we corrected/changed the manuscript in the following ways:

Reviewer 1:

#Table 1:

a) “Presurgical diagnosis of multifocality and bilaterality, number of foci...”. Thank you very much for this remark. Clearly, the presurgical diagnosis of PTMC before surgery is sometimes difficult but very important. To establish the most accurate diagnosis of thyroid tumors before surgery in all patients, we performed ultrasonography and ultrasound-guided fine needle aspiration biopsy (UG-FNAB) of all suspected tumors, as indicated for this tumor type. If the results indicate two or more suspected malignant tumors, we performed UG-FNAB in all lesions. Occasionally, we make a cancer diagnosis in more than one thyroid tumor. Thus, in such cases, we know before surgery that the patient has multifocal bilateral PTMC. We added the following adequate explanation to the manuscript: “Preoperative thyroid ultrasonography, ultrasound guided-fine-needle aspiration biopsy (UG-FNAB) and cytological examinations were performed in all cases. All suspected lesions were biopsied, and on this basis, presurgical multifocality and bilaterality were established.”

b) We are immensely grateful for this remark. Indeed, we made a mistake when we assessed the patients according to TNM staging. According to the 8^th^ Edition of the TNM Classification, in cases of well-differentiated papillary thyroid cancers, patients with N1 aged 55 years or above at the time of diagnosis are assigned to stage II. Thus, we reassessed and corrected the results in the table. Thank you.

c) We decided to not present the clinical classification of lymphatic nodules cN (pathologically changed lymphatic nodules) because these data were not strict and did not influence the results of our analysis. However, if you feel it is necessary to include these data, we will gladly reassess the medical records of all patients and include this clinical observation.

#Table 2:

We performed separate statistical analyses of minETE, capsular invasion and vascular invasion (Tables 2, 3, and 4).

In the Materials and methods, we added information regarding the following:

a) Surgical treatment, CLN dissection:

“All patients included in the study underwent radical surgery (total thyroidectomy with central lymph node dissection).”

b) Ablation/radioiodine therapy: The study was performed in the Surgical Department. After radical surgery, we sent all patients for consultation and potential qualification for RAI therapy at an oncology center in a different place.

“All individuals diagnosed with PTMC after surgery were routinely sent to the Oncology Center in Gliwice (Poland) for consultation and possible adjuvant radioiodine (RAI) therapy. The patients with pN0 features did not receive adjuvant RAI therapy, and the individuals with pN1 features received a single dose of ablated RAI in therapeutic amounts. All patients agreed to strict follow-up and remained under observation.”

c) Recurrence:

“For the purpose of this study, we assessed DFS time as the length of time (months) after primary surgery during which the patient survived without any structural evidence of disease. A p-value less than 0.05 was considered statistically significant.”

d) Follow-up time (min, max, medium):

“The follow-up duration was a minimum of 12 months and maximum of 144 months, with a median interval of 70 months.”

Although our manuscript was reviewed by American Journal Experts as recommended by the journal (we attached the certificate), after making our point-by-point corrections based on the suggestions by the reviewers, we sent our revised manuscript once again to AJE for proofreading (we have attached the new certificate). Thank you.

Reviewer 2:

First, we thank you very much for the opinion that our manuscript contains some valuable data. This is a very important acknowledgement for us. Thank you. Regarding your remarks, we corrected the text as follows:

1) We agree that the title is misleading. We changed the title to “Risk Factors of Papillary Thyroid Microcarcinoma that Predispose Patients to Local Recurrence.”

2) We absolutely agree that the topic undertaken in our study is controversial and that we do not have data confirming that a more aggressive treatment of PTMC provides better survival for all patients. Indeed, we analyzed only the risk factors for local recurrence and not overall survival time. In our analysis, all patients free of local recurrence were still alive, but the time of observation may be too short. Thus, we changed our discussion to avoid misleading readers. We drew hasty conclusions that local recurrence shortens the overall survival time, and as we know, in PTMC, this finding is not clearly established. Thank you for this valuable remark.

3) We included the data showing how many patients had 1, 2, 3 or 4 risk factors in Table 1 and the supportive data set.

4) We agree that the discussion was not well written; thus, we have rewritten and re-edited the discussion according to your suggestions. Thank you.

5) Although our manuscript was reviewed by American Journal Experts as recommended by the journal (we attached the certificate), after making our point-by-point corrections based on the suggestions by the reviewers, we sent our revised manuscript once again to AJE for proofreading (we have attached the new certificate). Thank you.

Best regards,

Krzysztof Kaliszewski MD, PhD
